# FGF8 promotes colorectal cancer growth and metastasis by activating YAP1

**DOI:** 10.18632/oncotarget.2822

**Published:** 2014-11-26

**Authors:** Rui Liu, Shan Huang, Yunlong Lei, Tao Zhang, Kui Wang, Bo Liu, Edouard C. Nice, Rong Xiang, Ke Xie, Jingyi Li, Canhua Huang

**Affiliations:** ^1^ State Key Laboratory of Biotherapy/Collaborative Innovation Center of Biotherapy, West China Hospital, Sichuan University, Chengdu, P. R. China; ^2^ State Key Laboratory of Oral Diseases, West China Hospital of Stomatology, Sichuan University, Chengdu, P. R. China; ^3^ Department of Biochemistry and Molecular Biology, and Molecular Medicine and Cancer Research Center, Chongqing Medical University, Chongqing, P. R. China; ^4^ The School of Biomedical Sciences, Chengdu Medical College, Chengdu, P. R. China; ^5^ Department of Biochemistry and Molecular Biology, Monash University, Clayton, Victoria, Australia; ^6^ School of Medicine, Nankai University, Tianjin, P.R. China; ^7^ Department of Oncology, Sichuan Provincial People’s Hospital, Chengdu, P. R. China

**Keywords:** FGF8, colorectal cancer, growth, metastasis, YAP1

## Abstract

Colorectal cancer (CRC) is a major cause of cancer-related death worldwide. The poor prognosis of CRC is mainly due to uncontrolled tumor growth and distant metastases. In this study, we found that the level of FGF8 was elevated in the great majority of CRC cases and high FGF8 expression was significantly correlated with lymph nodes metastasis and worse overall survival. Functional studies showed that FGF8 can induce a more aggressive phenotype displaying epithelial-to-mesenchymal transition (EMT) and enhanced invasion and growth in CRC cells. Consistent with this, FGF8 can also promote tumor growth and metastasis in mouse models. Bioinformatics and pathological analysis suggested that YAP1 is a potential downstream target of FGF8 in CRC cells. Molecular validation demonstrated that FGF8 fully induced nuclear localization of YAP1 and enhanced transcriptional outcomes such as the expression of CTGF and CYR61, while decreasing YAP1 expression impeded FGF-8–induced cell growth, EMT, migration and invasion, revealing that YAP1 is required for FGF8-mediated CRC growth and metastasis. Taken together, these results demonstrate that FGF8 contributes to the proliferative and metastatic capacity of CRC cells and may represent a novel candidate for intervention in tumor growth and metastasis formation.

## INTRODUCTION

Although the increased acceptance of colonoscopy, which allows for the removal of precancerous lesions, has led to a decline in the incidence of colorectal cancer (CRC), it remains the third most commonly diagnosed types of cancer and the fourth leading causes of cancer death for both men and women worldwide[1, 2]. Globally, mortality attributable to CRC is approximately half that of its incidence and 8% of all cancer deaths[3]. CRC survival is related to the stage of disease at diagnosis, with over 90% 5-year survival rate for cancers identified at an early stage; 70% with regional spread to less than 10% for patients with metastatic disease[3, 4]. Understanding of the molecular mechanisms of the disease in individuals at high risk of rapid tumor growth and progression is important for improved CRC prevention and control.

The human fibroblast growth factor (FGF) family consists of at least 23 different members that can be broadly grouped according to their affinity for FGF receptors (FGFRs)[5-9]. FGFs can act as mitogens, morphogens, and inducers of angiogenesis, and are required for many critical processes in the development of diverse tissues and organs from the earliest stages[5-9]. With such fundamental embryonic and homeostatic roles, FGFs are expressed in almost all tumor tissues[5-9]. For example, FGF1, FGF2, FGF6, FGF9 and FGF17 were overexpressed in prostate cancer, while FGF3 overexpression was observed in non-small-cell lung carcinoma[7-10]. The role of the FGF family has been also widely studied during tumor growth and metastasis and has been shown to induce EMT and increase the proliferative, motility and invasiveness of a variety of cell types[5, 6, 11]. For example, FGF1, FGF7 and FGF10 can induce EMT in bladder carcinoma cells[7]. Recently, FGFs have been shown to be involved in the progression of CRC. Elevated expression of FGF9, FGF10, FGF18, FGF-23 and FGFR2IIIc was observed in CRC, and expression of FGF9 and FGFR2IIIc negatively correlated with patients’ survival[9, 12-16]. In a previous study, we also demonstrated that FGFR4 promoted stroma-induced EMT in CRC and controls CRC cell metastasis *in vivo*[17]. However, the role of other FGFs in CRC, including FGF8, remains unclear.

FGF8 was originally identified as an androgen-induced growth factor from the conditioned medium of the mouse mammary carcinoma cell line SC-3[18, 19]. FGF8 is rarely detected in normal adult tissues, but widely expressed during embryonic development and in several forms of hormonal cancer including human breast, ovarian and prostate cancer[19-23]. FGF8 has been shown to mediate embryonic epithelial to mesenchymal direction and a mesenchymal to epithelial differentiation during embryonic development and is involved in gastrulation, early differentiation and organogenesis of brain, limbs and kidney[19, 22, 23]. High levels of FGF8 expression in clinical samples is associated with tumor progression and a poor prognosis in several cancers, including prostate and breast cancer[19-21, 24, 25]. In cell culture and transgenic animal models, FGF8 facilitates breast, prostate and ovarian cancer tumorgenesis, and increases tumor growth and angiogenesis by autocrine and paracrine loops[19, 26-29]. FGF8 is also known to confer an aggressive transformed phenotype to several cancer cells[19, 29]. For example, FGF-8 can enhance the invasive and migratory capacity of prostate cancer cells *in vitro* and promote bone metastasis *in vivo*[19, 29, 30]. In mouse mammary tumor cells, overexpression of FGF8 can induce EMT and anchorage independent growth *in vitro* and accelerated tumor growth *in vivo*[19, 31].

The Hippo signaling pathway was initially defined as a major regulator of tissue growth and organ size from genetic studies in Drosophila melanogaster[32-35]. Most upstream components in the Hippo pathway are evolutionarily conserved and serve as tumor suppressors in mammals[32-35]. The mammalian Hippo pathway comprises Yes-associated protein 1 (YAP1), Large tumor suppressors 1 and 2 (Lats1/2), Mammalian STE-20 kinases 1 and 2 (Mst1/2) and Mspone-binder (MOB1)[32-35]. YAP1, a nuclear transcriptional co-activator, binds to several transcription factors, such as ErbB4, SMAD, RUNX, TBX5, p73 and TEAD1-4, regulating the expression of diverse genes which are involved in the control of cell proliferation, apoptosis and movement[33, 34, 36-38]. Mst1/2-mediated Lats1/2 activation can negatively regulate the function of YAP1 by inducing phosphorylation of YAP1 on Ser 127 and Ser 358[33, 34, 37]. YAP1 amplification has been described as an essential oncogene in a large number of human cancers, including gesophageal squamous cell carcinomas, hepatocellular carcinomas, non-small cell lung cancer, prostate cancer, ovarian cancer and CRC[33, 34, 36, 39, 40]. For example, transgenic mice with YAP1 over-expression or knock-out of Hippo pathway genes show liver overgrowth with the eventual development of hepatic tumors[41], while YAP1 ectopic expression in cultured cells promotes cell growth and oncogenic transformation by activating TEAD-mediated transcription of the cell proliferation gene connective tissue growth factor (CTGF)[42, 43]. In addition, YAP1 was shown to be under-expressed in normal intestine, but highly expressed in CRC[44-46].

In the present study, we show that FGF8 is overexpressed in advanced CRC and promotes proliferation and metastasis of CRC cells by activating YAP1, suggesting FGF8 is a potential therapeutic target in CRC.

## RESULTS

### FGF8 is overexpressed in human CRC

**Figure 1:**
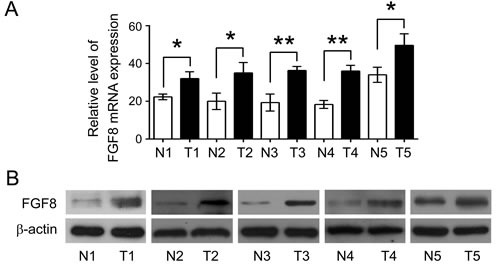
FGF8 is overexpressed in human CRC QRT-PCR (A) and imunoblot (B) analysis of FGF8 level in human CRC tissues (T) and adjacent normal mucosa tissues (N) from the same patient. All data were from at least three independent experiments. *, P<0.05; **, P<0.01.

**Figure 2:**
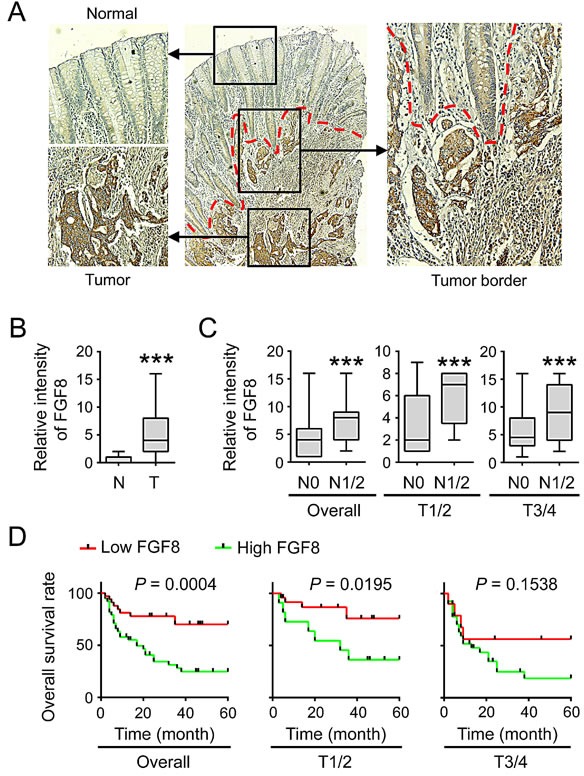
Overexpression of FGF8 correlates with lymph node metastasis and poor prognosis in CRC (A) Immunohistochemical staining of FGF8 in tumor and corresponding colorectal mucosa. (B) Immunohistochemical scores for FGF8 in normal colorectal mucosa and CRC tissues. (C) Expression of FGF8 in the primary tumors without (N0) or with (N1/N2) lymph node metastasis was analyzed. Left, overall tumors; middle, stage T1–T2; right, stage T3–T4. (D) Kaplan–Meier survival curves of CRC patients with low (n = 34) and high (n = 38) FGF8 expression. Left, overall tumors; middle, stage T1–T2; right, stage T3–T4. ***, P<0.001.

To determine the expression pattern of FGF8 in human colorectal tissues, paired non-tumor and tumor tissues (n = 5) from frozen tissue samples were analyzed by qRT-PCR and immunoblot analysis. FGF8 expression was found to be overexpressed in CRC tissues compared with adjacent non-tumor tissues at both the mRNA and protein levels (Figure 1A and 1B). Immunohistochemistry staining was further performed on a panel of 98 colorectal cancer specimens and 42 matched adjacent normal colorectal mucosa specimens to investigate the potential clinical role of FGF8 in CRC. As shown in Figure 2A, strong FGF8 staining was mainly observed in the cytoplasm of tumor cells, while weak FGF8 expression was detected in the proliferative zone of colorectal epithelium in normal colorectal tissue, but no FGF8 expression was detected in superficial colorectal epithelial cells. FGF8 positive staining was observed in 99% (97/98) of CRC tissues compared to 42% (18/43) of normal mucosa tissues. The staining intensity for FGF8 in tumor cells was significantly higher than in normal mucosal epithelial cells (Figure 2B). These results demonstrate that FGF8 is overexpressed in CRC.

### Elevated FGF8 is associated with lymph node metastasis and poor survival in CRC patients

We next analyzed the relationship between FGF8 expression in tumor tissues and the clinic-pathological parameters of the 97 CRC patients. The results showed that FGF8 expression was not associated with patient age, sex or tumor size (data not shown), but was significantly associated with lymph node metastasis. In both early-stage (T1/2) and late-stage (T3/4) colorectal carcinoma, FGF8 expression was much higher in the primary CRC tissue from individual patients with metastatic lymph nodes compared to those without metastatic lymph nodes, suggesting FGF8 is involved in metastasis of CRC (Figure 2C).

Moreover, FGF8 levels were also prognostic for overall survival (OS). A Kaplan-Meier survival analysis showed that subjects with high FGF8 expression had a significantly shorter 5-year OS time compared to those subjects with low FGF8 expression (log-rank test, P < 0.001, Figure 2D, left). Furthermore, a high level of FGF8 expression was more likely to be associated with poor outcome in patients with T1/2stage colorectal carcinoma (Figure 2D, middle) compared to those with T3/4 stage disease (Figure 2D, right). In a univariate analysis examining clinic-pathologic prognostic variables, the expression of FGF8 was significantly correlated with overall survival. Factors showing significance by univariate analysis were adopted in multivariate Cox proportional hazards analysis. The result showed that FGF8 acted as a potential prognostic marker for predicting patient outcome.

### FGF8 promotes an aggressive phenotype in CRC cells

**Figure 3:**
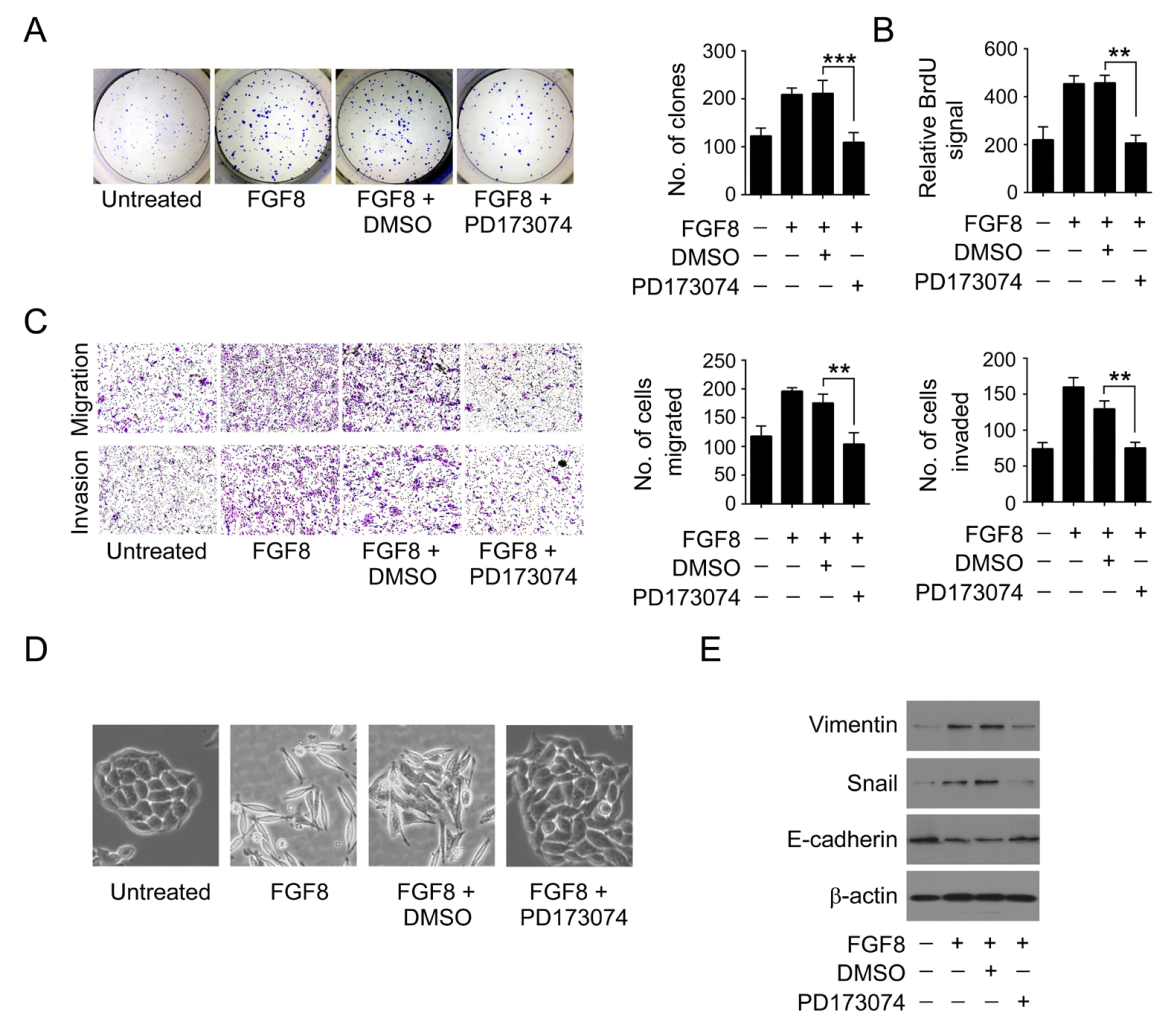
FGF8 promotes an aggressive phenotype in CRC cells RKO cells were treated with or without FGF8 and/or PD173074. (A) Representative photographs of colony formation 14 days after culture of cells. (B) Proliferation rate as measured by BrdU labeling for 12 hours. (C) Quantitative analysis of cell migration and matrigel invasion assays. Migration was analyzed at 24 h, and invasion at 48 h. (D) Representative phase-contrast images of RKO cell morphology. (E) Expression of Snail, E-cadherin and Vimentin was examined by immunoblot. All data were from at least three independent experiments. **, P<0.01; ***, P<0.001.

To determine the potential significance of FGF8 in colorectal cancer progression, the proliferative, migratory and invasive capacities of RKO cells were compared in the presence or absence of FGF8. As shown as Figure 3A-C, FGF8 produced about 1.8-fold more colonies in the colony formation assay (Figure 3A) and 2-fold augmentation of BrdU labeling (Figure 3B), induced RKO cells migration by approximate 1.6-fold and increased the invasion potential as demonstrated by matrigel invasion by more than 2-fold (Figure 3C). These effects were inhibited after treatment of cells with a pan FGFR inhibitor, PD173074 (Figure 3A-C). To rule out the potential cell type specific effect, we further examine the role of FGF8 on other two CRC cell lines, SW480 and HCT116. As expected, FGF8 treatment also significantly enhanced the proliferative, migratory and invasive ability of both SW480 and HCT116 cells (Figure S1). These results demonstrate that FGF8 promotes an aggressive phenotype in CRC cells.

FGF8 has been commonly studied during developmental and pathological EMT, which is widely considered to contribute to cancer metastasis[19]. Therefore, it was of particular interest to examine whether there was an involvement of FGF8 in the EMT process of CRC cells. The results showed that FGF8 treatment induced a mesenchymal phenotype in RKO cell line (Figure 3D). Further, FGF8 treatment also reduced expression of the epithelial marker E-cadherin and increased the levels of mesenchymal markers Vimentin and Snail in RKO, SW480 and HCT116 cells, and that these effects could also be abrogated by PD173074 (Figure 3E and Figure S2), suggesting FGF8 induces a malignant phenotype by promoting EMT in CRC cells.

### FGF8 increases tumor growth and metastasis in mice

**Figure 4:**
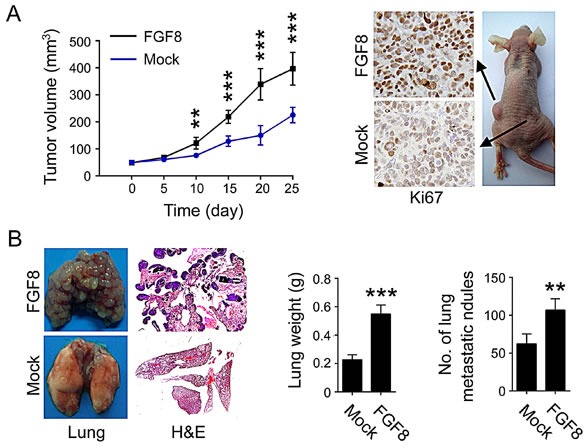
FGF8 promotes tumor growth and metastasis in mice (A) Mean tumor volume and Ki67 expression in tumors after subcutaneous transplantation of RKO-FGF8 or RKO-mock cells. (B) Histopathology showing the lung metastases in mice and quantification of the lung weight and number of metastases following tail-vein injection with RKO-FGF8 or RKO-mock cells. **, P<0.01; ***, P<0.001.

To study the effect of FGF-8 on tumor growth and metastasis *in vivo*, RKO cells stably expressing FGF-8 (RKO-FGF8) or mock vector (RKO-mock) were subcutaneously or intravenously injected into nude mice to assess local tumor growth and metastasis, respectively. As shown as Figure 4A, the growth of those tumors formed by RKO-FGF8 cells following subcutaneous injection was much faster than control tumors, indicated by increased tumor volume and expression of Ki67. In the mouse lung metastatic assay, the weight of the lungs following injection of RKO-FGF8 cells was markedly increased compared with injection of control cells. Additionally, the average number of metastases in mice lungs derived from RKO-FGF8 cells was 1.5-fold greater than control cells as determined by H&E staining (P < 0.01; Figure 4B). These results show that FGF8 has a profound impact on the tumor growth and formation of metastases by the CRC cells.

### Construction of the global PPI network

**Figure 5:**
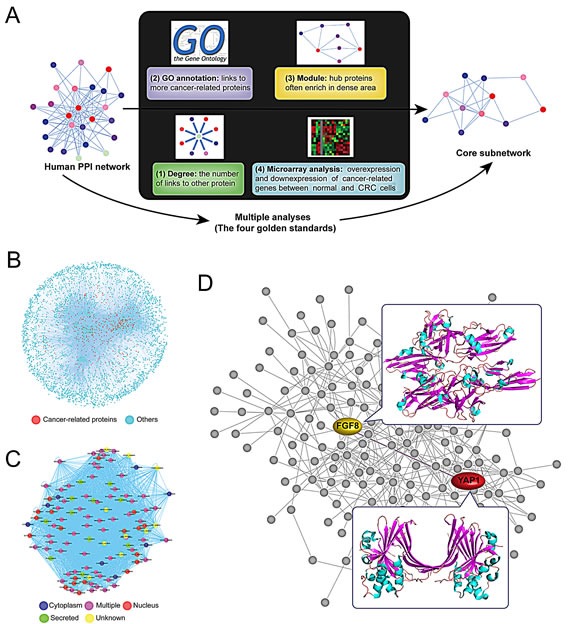
Multiple analyses of hub proteins and identification of FGF8-YAP1 interaction in CRC cells (A) We identified hub proteins implicated in core pathways according to the following four gold standards: degree, link, module and microarray analyses. (B) The global PPI network. (C) The core sub-network in cancer. (D) Identification of FGF8-YAP1 interaction in CRC cells.

To explore the molecular mechanisms underlying FGF8-induced proliferation and metastasis of CRC cells, we computationally constructed a global human PPI network, covering almost all PPIs from IntAct, HPRD, HomoMINT, BOND and BioGRID. Owing to this mathematics model, we identified 2,110 apoptosis-related proteins (GO: 0006915) from GO database. To construct the set of true-positive gene pairs, physical protein-protein interactions were derived from manually created PPI databases, including 37,710 from BioGRID among 8,982 proteins, 8,044 from BOND among 4,073 proteins, 14,892 from HomoMINT among 6,240 proteins, 39,044 from HPRD among 9,619 proteins, and 34,935 from IntAct among 8,849 proteins. A total number of 85,083 unique PPIs addressing 13, 128 proteins were prepared as a data source for our Gold Standard Positive (GSP) set by integrating PPIs from online databases (Figure 5A). Proteins located in plasma membrane seldom interact with those in nucleus; therefore, we generated a Golden Standard Negative (GSN) set that could be defined as all the possible pair-wise combinations, in which one protein is assigned to the plasma membrane and the other to the nucleus according to GO cellular component annotation, resulting in 23,169,177 pairs in our GSN. Additionally, there are 25,620 and 204,919,890 protein pairs in the STS and raw predicted dataset.

According to the two golden sets, we integrated four different types of biological datasets and chose the likelihood ratio (LR=117) as the reliability of individual dataset for inferring the PPIs. Each dataset could be divided into several bins due to their intrinsic characters, and LR for each bin was calculated, indicating the corresponding results of cross-species interolog mapping, gene co-expression profiles, domain-domain interaction (DDI) and smallest shared biological process (SSBP), respectively. Subsequently, we used LR cutoff as 113 and achieved the global PPI network with 12,809 binary PPIs by combining the prediction set and the positive set. Before using the lunched Naive Bayesian model, STS containing 12,809 interacting protein pairs conformed by 4,818 unique proteins was inputted the network model, resulting the area under ROC curve.

### Multiple analyses of hub proteins and identification of FGF8-YAP1 interaction in CRC cells

In this study, we identified hub proteins implicated in core pathways according to the following four golden standards (Figure 5A). Firstly, the degree of each protein in the function-related network is calculated as the number of links that one protein possesses to the other, where high-degree proteins tend to play a more important role in the network. Thus, we selected the number of degrees which is bigger than or equal to 300 as our standard for identifying candidate hub proteins.

Secondly, we ensure that hub proteins connect more cancer-related proteins that have been annotated by GO than other (non-hub) proteins, thereby giving them a particular focus when developing novel cancer targets. We choose the number of links to other known cancer-related proteins that are bigger than or equal to 300 (the standard of classical hub proteins) or 200 (the standard of novel hub proteins) respectively as our standard for further filtering candidate hub proteins. Thirdly, we suggest that the network module is crucial for helping to identify hub proteins because they typically enrich in the “dense area” rather than “sparse area” in cancer. Thus, we found a few of conserved modules that could enrich more candidate hub proteins into the network.

Fourthly, since significance analysis of microarrays (SAM) analysis is performed on data from expression microarray to identify genes with greatly divergent expressions between normal and CRC cells, we indicated that the proteins, identified as divergent expression proteins that were extracted as functional hub proteins, are dependent on gene co-expression profile.

As a result, we computationally constructed the global human PPI network, and further modified it into the core network. The four above-mentioned gold standards which can be integrated into an appropriate approach to decrease the false-positive PPIs were used in this study. Furthermore, cancer-related hub proteins in the global PPI network (Figure. 5B) were classified by their different subcellular localizations (Figure. 5C). Interestingly, we found that FGF8 plays a key role in this sub-network, interacting with other hub proteins, such as YAP1, in the context of CRC cells (Figure. 5D).

### FGF8 can activate YAP1 signaling in CRC cells

**Figure 6:**
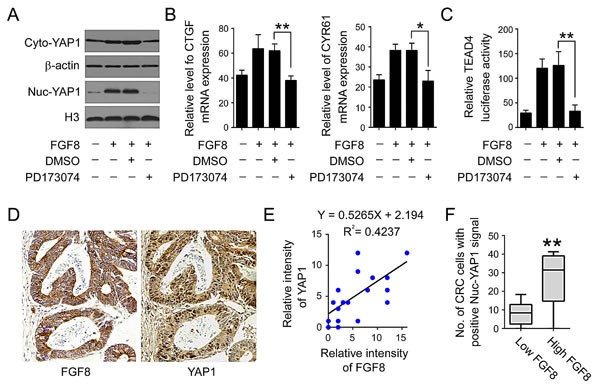
FGF8 activates YAP1 signaling in CRC cells (A) Immunoblot analysis of cytoplasmic and nuclear YAP1 in RKO cells treated with or without FGF8 or/and PD173074. β-Actin was used as a cytoplasmic protein loading control, and histone-3 (H3) was used for nuclear protein loading control. (B) MRNA level CTGF and CYR61 was examined by qRT-PCR. (C) Transcription activity of TEAD4 was examined by luciferase assay. (D) Expression of YAP1 and FGF8 in serial human colorectal tumor sections was examined by immunohistochemical staining. (E) Correlation between the expression levels of YAP1and FGF8. (F) Expression of YAP1 in high-FGF8-expressing tumors and low-FGF8-expressing tumors was analyzed. All data were from at least three independent experiments.*, P<0.05; **, P<0.01.

Yes-associated protein 1 (YAP1), a downstream transcriptional co-activator of the Hippo pathway, is a major regulator of organ size by regulating cell proliferation and survival in vertebrates[32-34]. As such, YAP1 can act as an oncogene and is amplified in various adult carcinomas including CRC[32-34]. Further, YAP1 interaction with transcription factors such as TEAD1-4 in the nucleus can promote cancer cell proliferation, anchorage-independent growth, EMT and metastasis[32-34, 43]. We therefore assessed the potential impact of FGF8 on YAP1 in CRC cells. As indicated as Figure 6A and S3A-B, elevated expression of YAP1 was observed in both the nucleus and cytoplasm of FGF8-treated RKO, SW480 and HCT116 cells. Further, FGF8 could also induce the expression of YAP1 target genes, CTGF and CYR61, and the transcription activity of TEAD4, a target transcription factor of YAP1, while the FGFR inhibitor PD173074 can abolish these effects (Figure 6B–C and S3C-F). To explore whether YAP1 is also correlated with FGF8 in human colorectal tissues, levels of YAP1 were analyzed in 20 resected colorectal cancer specimens, which were also examined with an antibody to FGF8. In CRC tissues, YAP1 expression was mainly observed in the nuclear of tumor cells (Figure 6D). Nuclear-YAP1 and FGF8 levels were plotted against each other, and the staining of nuclear YAP1 in high-FGF8-expressing tumors was stronger than that in low-FGF8-expressing tumors, suggesting that the expression of YAP1 is associated with FGF8 level in colorectal cancer (Figure 6E-F). These results demonstrate that FGF8 activates YAP1 signaling in CRC cells.

### YAP1 is essential for FGF8-mediated CRC malignant progression

**Figure 7:**
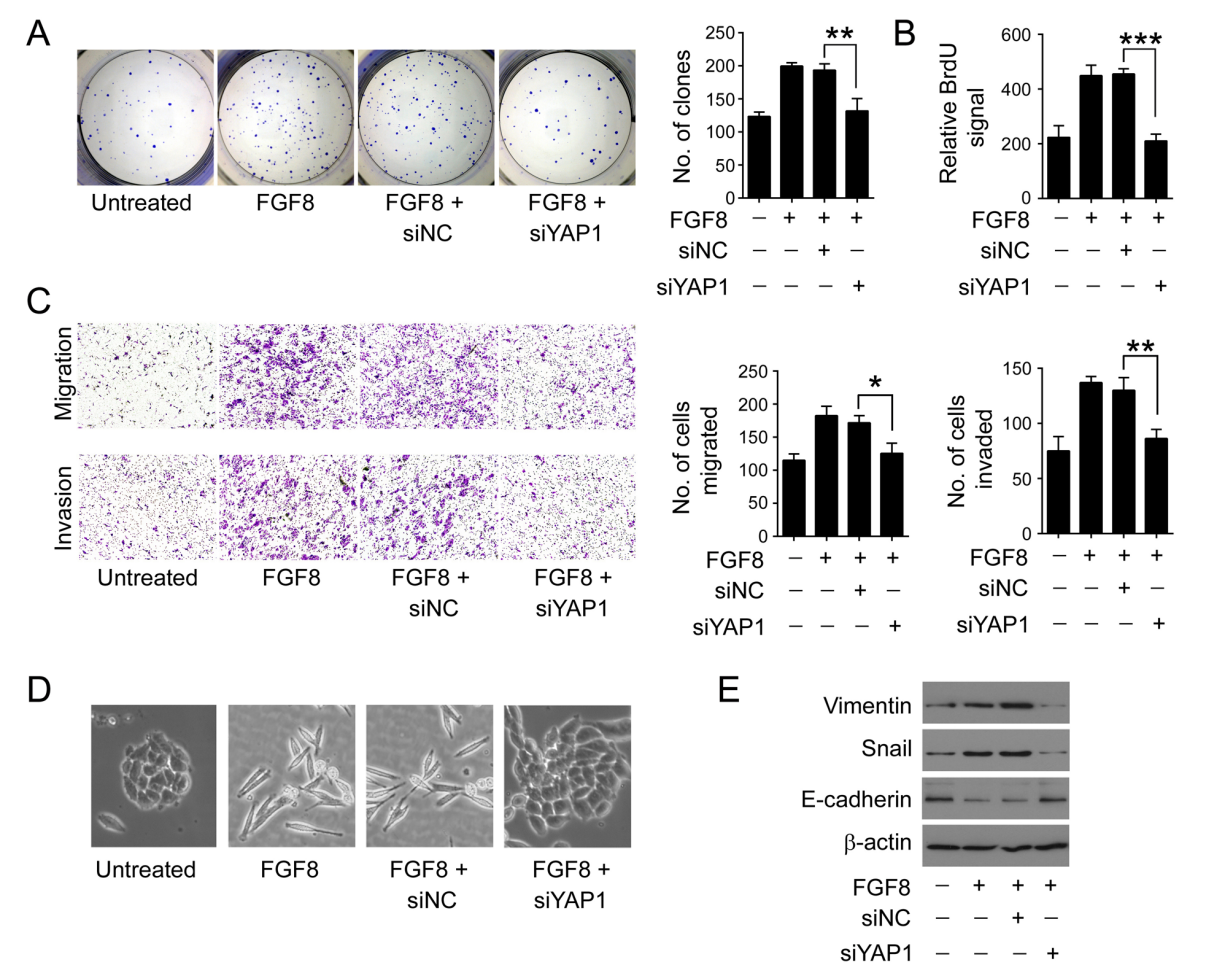
YAP1 is essential for FGF8-mediated tumor growth and metastasis FGF8-treated RKO cells were transfected with siYAP1 or siNC. (A-B) Proliferative activity was measured by a colony formation assay (A) and BrdU labeling (B). (C) Cell migration and invasion was examined by transwell assay and matrigel invasion assays. (D) Representative phase-contrast images of RKO cell morphology. (E) Expression of Snail, E-cadherin and Vimentin was examined by immunoblot. All data were from at least three independent experiments. *, P<0.05; **, P<0.01; ***, P<0.001.

To determine whether YAP1 was required for the FGF8-induced aggressive phenotype in CRC cells, YAP1 expression was knocked down in FGF8-treated RKO cells (Figure S4). As indicated as Figure 7A and B, suppression of YAP1 reduced colony number and BrdU incorporation induced by FGF8. Additionally, it counteracted FGF8-induced cell migration and invasion in RKO cells (Figure 7C), accompanied with reversion to a more compact epithelium-like morphology(Figure 7D). Correspondingly, loss of YAP1 increased expression of the epithelial marker E-cadherin and reduced the levels of mesenchymal markers Vimentin and Snail in FGF8-treated CRC cells (Figure 7E). Similar effects were also observed in SW480 and HCT116 cells (Figure S5 and S6). These results indicate that YAP1 contributes to the FGF8-induced proliferative and metastatic capacity of CRC cells.

### FGF8 activates YAP1 signaling through increasing the transcription of YAP1

The Hippo pathway restricts the transcriptional co-activation capacity of YAP1 by phosphorylating it for cytoplasmic localization and protein degradation. Conversely, activation of YAP1 is usually though inhibition of Lats activity [32-34]. Thus, we next examined the involvement of canonical Hippo signaling in FGF8-induced activation of YAP1. As expected, silencing Lats1/2 can activate YAP1, indicated by nuclear translocation of YAP1, enhanced transcription of CTGF and CYR61, and increased transcriptional activity of TEAD4 (Figure S7). But interestingly, even when Lats1/2 was knocked down, FGF8 can further enhance the protein level of both total and nuclear YAP1, the mRNA level of CTGF and CYR61, as well as the transcriptional activity of TEAD4, suggesting inhibiting degradation through Hippo pathway is not the only cause for FGF8-induced YAP1 accumulation(Figure S7). Therefore, we further attempt to determine whether FGF8 regulates the transcription of YAP1. The results showed that FGF8 treatment obviously increased the YAP1 mRNA levels in all three CRC cell lines, and PD173074 can inhibit this accumulation, suggesting FGF8 can enhance the transcription of YAP1.

## DISCUSSION

Although significant advances have been made in diagnostic, surgical and therapeutic techniques, the prognosis for patients with advanced or recurrent states of CRC remains dismal[1-4]. Thus, discovery of a sensitive and specific diagnostic biomarker for detection of individuals with rapid tumor growth and progression, and introduction of novel targeted therapeutic drugs are matters of pressing concern. To improve the survival rates of patients with advanced stage disease, the detailed molecular mechanisms underlying initiation and progression of CRC must first be thoroughly understood. The FGF/FGFR family is known to be widely involved in many physiological and pathological processes including embryonic development, repair, tumor growth and progression in an autocrine or paracrine manner[5-9]. FGFs exert biological effects as potent growth factors for inducing proliferation and differentiation in primary epithelial cells, which makes FGF signaling susceptible to be hijacked by cancer cells[5-9]. Accumulating evidence has linked carcinogenesis in a range of tissue types with the dysregulation of FGF signaling, including control of cancer cell proliferation, modulation of tumor cell adhesion and migration, and support of neoangiogenesis[5-9]. A high percentage of CRCs overexpress a number of FGFs and FGF receptors, including FGF-1, FGF-2, FGF-3, FGF-7, FGF-9, FGF-10, FGF-18, FGF-19, FGF-20, FGF23 and FGFR1-4[11][12-16, 47-50]. For example, Sonvilla*et al.* showed that FGF18 was progressively enhanced during colon carcinogenesis reaching very high levels in carcinomas and affecting both tumor cells and the tumor microenvironment in a pro-tumorigenic and pro-metastatic way[50]. SATO *et al* also demonstrated a relationship between overexpression of FGFR1 and liver metastasis in colorectal cancer[49]. In this current study, mild immunoreactivity for FGF8 was observed in colorectal cancer cases, and is significantly correlated with lymph node metastasis and poor prognosis (Figure 1 and 2).

FGF8 regulates a range of physiological processes such as limb formation, central nervous system development, left–right axis establishment, angiogenesis and wound healing, as well as pathological routes to tumorigenesis[19, 22, 23]. FGF-8 is widely expressed in developing tissues in a temporally and spatially regulated manner, but has a strictly restricted expression pattern in a limited number of normal adult tissues, such as certain cell types involved with spermatogenesis and oogenesis[19, 22, 23]. There have been no reports about FGF8 in CRC, but aberrant expression of FGF8 has been observed in several other cancers, especially in hormone-responsive tumors such as prostate and breast cancer[8, 19, 24, 51]. In prostate and breast cancer, the overexpression of FGF8 is correlated with advanced tumor stage and shorter survival times[8, 19, 20, 24, 25]. Transgenic expression of FGF8 in mice can induce mammary and salivary gland tumors as well as development of ovarian stromal hyperplasia[19, 28]. Engineered overexpression of FGF8 in both prostate and breast cancer cell lines has been shown to be tumor promoting in many *in vitro* and *in vivo* studies[8, 19, 25, 26]. For example, the overexpression of FGF8 in prostate cancer LNCaP cells and mammary tumor MCF-7 cells enhanced growth and invasion *in vitro* and promoted tumor growth *in vivo*[25, 29, 52, 53]. Additionally, Valta*et al* found expression of FGF-8 in PC-3 prostate cancer cells increased their growth as intratibial tumors and markedly affected formation of bone lesions in this *in vivo* model of prostate cancer metastasis[30]. Here, we report that FGF8 treatment accelerated the growth rate, increased both clonogenic and invasive activity *in vitro*, and similarly, overexpression of FGF8 facilitated *in vivo* tumorigenicity and metastasis of CRC cells, suggesting that FGF8 plays an important role in CRC progression (Figure3,4 and S1). Furthermore, during early embryonic development, FGF8 has been shown to mediate EMT, which has been noted as a critical event in the late stages of tumor progression[19]. Key steps in tumor-associated EMT are down-regulation of E-cadherin by transcriptional repressors such as Snail1, ZEB1, and Twist, and induction of mesenchymal-specific gene expression, such as Vimentin, Fibronectin, and N-cadherin, which leads to the conversion of stationary epithelial cells into migratory mesenchymal cells[11, 12]. In this study, we also found that FGF8 can induce a fibroblastic change in RKO cell morphology, with altered EMT-specific gene expression, including repression of E-cadherin and activation of Snail and Vimentin, indicating that FGF8 contribute to CRC metastasis by inducing EMT (Figure 3, Figure S2).

To explore the molecular mechanism underlying FGF8-induced proliferation and metastasis in CRC, we analyzed the protein-protein interaction network in CRC cells by bioinformatics and found YAP1 was a potential downstream molecule of FGF8 (Figure 5). Pathological data also demonstrated that the nuclear expression of YAP1 is positively correlated with FGF8 level in clinical CRC samples (Figure 6D-F). YAP1, a transcriptional co-activator, is inhibited by the Hippo tumor suppressor signaling pathway and regulates multiple cellular processes by activating several transcription factors, such as TEAD1-4[32-38, 42, 54]. YAP1 plays a critical role in organ growth and has been suggested to be a candidate human oncogene in multiple tumors[33-35, 39, 41, 42, 54]. Since YAP1 is mainly involved in regulating the transcriptional outcome to govern cell proliferation and survival, it can be hijacked by cancer cells to facilitate their own growth, including induction of cancer stem cells and metastatic colonization[33-35, 39, 42, 43, 55]. The up-regulation and nuclear localization of YAP1 has been shown to correlate with progression, metastasis and poor patient outcome in several cancers, such as non small cell lung cancer, breast cancer, gastric carcinoma and hepatocellular carcinoma[32-34, 56-58]. Further, YAP1 overexpression in multiple cancer cell lines can promote proliferation, inhibit apoptosis and enhances *in vitro* invasive and metastatic capacity[33, 34, 38, 56, 59]. Recent studies also showed that YAP1 plays a pivotal role in the initiation and progression in CRC[44-46]. In this study, we found that FGF8 can induce activation of YAP1 signaling, and silencing of YAP1 reversed FGF8-induced proliferation, migration and invasion in CRC cells, suggesting YAP1 is important in the acquisition of an aggressive phenotype in FGF8-treated CRC cells (Figure 6-7 and S3-5). In addition, YAP1 is also implicated in EMT[38, 43]. For example, over expression of YAP1 mutants that cannot be phosphorylated, can overcome cell contact inhibition and contribute to metastatic properties associated with expression of EMT markers in MCF10A mammary epithelial cells[42, 43]. Further, Shao *et al* showed KRAS and YAP1 converge on the transcription factor FOS and activate a transcriptional program involved in regulating EMT by inducing expression of Vimentin and Slug in CRC HCT116 cells[60]. Here, we also found that YAP1 was essential for FGF8-mediated EMT in RKO, SW480 and HCT116 cells (Figure 7 and S6). More interestingly, cell–cell contact, which can restrict cell proliferation and migration, has been shown to trigger the Hippo pathway leading to phosphorylation of YAP1 and thereby inhibiting its ability to stimulate proliferation and oncogenic transformation[32-34]. Thus, YAP1-induced EMT may induce loss of cell–cell contact leading to the inhibition of Hippo signaling and further accumulation of YAP1 in the nucleus of CRC cells.

**Figure 8:**
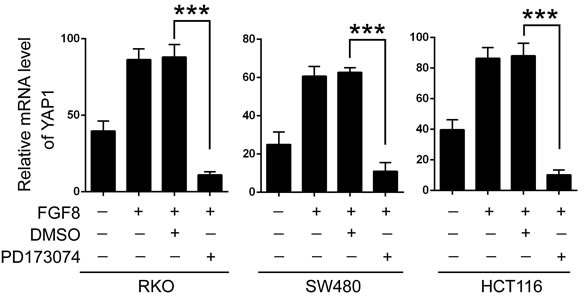
FGF8 activates YAP1 signaling through increasing the transcription of YAP1 QRT-PCR analysis of YAP1 mRNA level in FGF8-treated RKO, SW480 and HCT116 cells in the present or absent of PD173074.

Restricting the activity of YAP1 is critical for maintaining tissue homeostasis[32-34]. Under physiological conditions, YAP1 is phosphorylated and inhibited by Lats kinases, which are the core components of the Hippo pathway. Lats-mediated phosphorylation on Ser127 promotes YAP1 binding to 14-3-3 proteins and consequently its cytoplasmic retention, while phosphorylation on Ser381 catalyzes YAP1 ubiquitination, ultimately leading to YAP1 degradation[33, 34, 37, 54]. Besides Hippo signaling, several other signaling pathways have been also shown to induce activation of YAP1, such as WNT, integrin, Rho/Rac, Notch, TGF-β and GPCR signaling, which are all also downstream signaling pathways of FGFs[55, 61-67]. For example, Rosenbluh *et al* showed that YAP1 was an attractive target in β-catenin-driven cancers, while FGF8 can accelerate mammary carcinogenesis in MMTV-Wnt1 transgenic mice[61, 64]. In present data, in addition to inhibition of Lats1/2, FGF8 can further enhance YAP1 expression by promoting YAP1 transcription, suggesting that increased transcription of YAP1isan important cause in FGF8-induced activation of YAP1 signaling (Figure 8 and S7).

In conclusion, we have demonstrated that overexpression of FGF8 is correlated with metastasis and poor prognosis in patients with CRC, and elevated FGF8 can activate YAP1 signaling, which in turn, induces EMT and increases growth, invasion and lung metastases in experimental CRC cell lines and tumors. Our results underscore the clinical potential of FGF8 for the early detection or therapeutic treatment of advanced CRC.

## MATERIAL AND METHODS

### Clinical specimens

All colorectal carcinomas and corresponding adjacent normal tissues were obtained from Sichuan Provincial People’s Hospital (Chengdu, China). Tumor stage was determined according to the TNM classification system of the International Union against Cancer (UICC) [68]. Tumor differentiation was graded using Edmondson Steiner grading by two experienced pathologists. The clinicopathologic characteristics of 98 patients are summarized in Table 1. Informed consent for tissue procurement was obtained from all patients or their relatives before study initiation, and Ethics approval was obtained from the Institutional Ethics Committee of Sichuan University.

**Table 1:** Clinico-pathologic parameters of colorectal cancer patients

Characteristics	Number (%)
Gender n= 98	
Male	58 (59.2%)
Female	40 (40.8%)
Age (years) n= 98	
<65	52 (53.1%)
≥65	46 (46.9%)
Tumor location n= 98	
Colon	79 (80.6%)
Rectum	19 (19.4%)
TNM T stagen= 98	
T1	16 (16.3%)
T2	35 (35.7%)
T3	35 (35.7%)
T4	12 (12.2%)
TNM N stage n= 81	
N0	58 (71.6%)
N1	16 (19.8%)
N2	7 (8.6%

### Immunohistochemistry

Slides were stained using the Envision System horseradish peroxidase method (DakoCytomation Inc., Carpinteria, CA) according to the manufacturer’s instructions. To estimate the score of each slide, at least eight individual fields were chosen, and 100 cancer cells were counted for each field. The score for each slide was measured as the cross product of the value of immunostaining intensity (A) and the value of proportion of staining-positive cells (B), as described previously. Immunostainingintensity was divided into five grades: 0, negative; 1, weak; 2, moderate; 3, strong; 4, very strong. The proportion of staining-positive cells was divided into five grades: 0, <5%; 1, 6 –25%; 2, 26 –50%; 3, 51–75%; 4, >75%. The results were defined as: 0-4, low; 5-16, high. Results were assessed and confirmed by two independent experienced pathologists[17].

### Cell culture

The RKO, SW480 and HCT116 cell line was purchased from American Type Culture Collection (ATCC, Rockville, MD). Cells were maintained in Dulbecco’s Modified Eagle’s Medium (DMEM, Gibco, USA) containing 10% fetal bovine serum (Hyclone, USA), penicillin (107 U/L) and streptomycin (10 mg/L) at 37 °C in a humidified chamber containing 5% CO_2_.

### Real time RT-PCR (qRT-PCR)

RNA was extracted using TRIzol reagent (Invitrogen) and cDNA was transcribed using Revert Aid^TM^ First Strand cDNA Synthesis Kit (Fermentas) according to the manufacturer’s instructions. Analysis was performed on a Bio-Rad CFX96 Real-Time PCR System (Bio-Rad) according to the manufacturer’s instructions.

### Reagents

PD173074 was purchased from Sigma and used at 1 μm. FGF8 recombinant protein was purchased from Protech and used at 250 ng/ml. YAP1-specific siRNA was purchased from Dharmacon, and the siRNAs targeting LAST1 or LAST2 were purchased from Sigma Aldrich. The following primary antibodies were used: rabbit-anti-E-cadherin (Abcam), rabbit-anti-Snail (Abcam), mouse-anti-Vimentin (Santa Cruz, Abcam), mouse-anti-FGF8 (Abcam), rabbit-anti-YAP1 (Abcam), rabbit-anti-LATS1 (Abcam), rabbit-anti-LATS2 (Abcam), rabbit-anti-Histone H3 (Abcam).

### Immunoblot

Cells were lysed with RIPA buffer (50 Mm Tris base, 1.0 mM EDTA, 150 mM NaCl, 0.1% SDS, 1% Triton X-100, 1% sodium deoxycholate, 1 mM PMSF). Proteins were separated on 12% or 15% SDS-PAGE, and transferred to PVDF membranes (Amersham Biosciences). After blocking with Tris-buffered saline (TBS) containing 0.1 % Tween 20 and 5% skimmed milk, blots were incubated with the respective primary antibodies for 2 h at room temperature and washed 3 times in TBS with Tween20. Subsequently, the blots were incubated with HRP-conjugated secondary antibody (diluted 1:10,000; Santa Cruz Biotechnology) 2 h at room temperature. Finally, the blots were visualized by enhanced chemiluminescence (Amersham Biosciences).

### BrdU labeling assay

The BrdU labeling assay was performed in 96 well plate format. BrdU was purchased from Roche Applied Science (Indianapolis, IN). After treatment, BrdU was added to a final concentration of 10 mM, and the cells were incubated for another 12 h. BrdU signal was measured by using 5-Bromo-2′-deoxy-uridine Labeling and Detection Kit III (Roche).

### Cell migration and invasion assays

Transwell 24-well chambers (Corning) were used for *in vitro* cell migration and invasion assays. For the cell migration assay, 2.5×10^4^ cells were seeded in the upper well of a transwell chamber. For the invasion assays, Matrigel (1:3, BD, USA) was added to the transwell chambers, and cells were seeded after incubation at 37 °C for 4 h. Cells on the upper side of the filter were removed after 24 h for the migration assay or 48 h for the invasion assay. The filter membrane was stained with crystal violet, and the number of the cells that remained adherent to the underside of the membrane were counted using an inverted microscope (Zeiss Axiovert).

### *In vivo* tumor proliferation and metastasis

All animals were humanely treated under the guidelines of the Institutional Animal Care and Treatment Committee of Sichuan University. For *in vivo* tumor proliferation assays, 5×10^7^ RKO cells stably expressing FGF8 or mock vector were transplanted subcutaneously into male athymic nude mice (5 mice per group). The tumor volumes were evaluated as follows: tumor volume (mm^3^) = (length × width^2^)/2. Animals were sacrificed 25 days after injection. Tumors were dissected and fixed in formalin for immunostaining with Ki67. For metastasis assays, 5×10^7^RKO-FGF8 or RKO-mock cells were injected into male athymic nude mice (4 mice per group) through the tail vein. Animals were sacrificed on day 35. The lungs were excised and fixed in formalin for standard hematoxylin and eosin (H&E) staining.

### Bioinformatics analyses

#### Retrieval of functional genomics data

Data were collected from Human Protein Reference Database (HPRD)[69], Biomolecular Object Network Databank (BOND)[70], IntAct[71], HomoMINT and BioGRID[72, 73] to build the global PPI network. All the data were preprocessed into pair-wise scores, reflecting the similarity between protein pairs, and Gold Standard Positive (GSP) interaction set was constructed using these online databases. Gold Standard Negative (GSN) interaction set was defined through protein pairs where one was a membrane protein (6,637 proteins) and the other a nuclear protein (4,138 proteins), as assigned by Gene Ontology (GO) Consortium. However, 404 proteins were removed because they were assigned to both components, and 23,169,177 unique pairs, in total, were identified except for 5,275 overlapping pairs with GSP. Additionally, the data in Standard Test Set (STS) were retrieved from Database of Interacting Proteins (DIP)[74] and matched randomly by these proteins, and apoptotic proteins were from GO annotation. Raw data were constructed by random matching amongst all the human proteins in UniProt database.

#### Multiple sources of biological data

##### Gene co-expression profiles

Proteins that can interact with each other often possess similar gene expression patterns; thereby, genes that can co-express should be more likely to interact than genes that cannot co-express. To identify genes that are co-expressed, microarray data from colorectal cancer and normal colorectal cells treated with 2.5 mM DTT were used to measure the pair-wise co-expression level of related genes [75]. The co-expression level is calculated as Pearson Correlation Coefficient ρ

$$\rho_{X,Y}=\frac{\sum_{i=1}^{n}\left(X_{i}-\bar{X})(Y_{i}-\bar{Y}\right)}{(n-1)\sigma_{X}\sigma_{Y}}$$

Where X and Y are expression level data vectors of length n for two genes, and are means, and σX and σY are the standard deviations.

##### Domain-domain interaction

Because physical associations between protein domains can mediate protein interactions, identifying the pairs of domains enriched amongst known interacting proteins is usually used to predict novel protein interactions. Thus, domain-domain interaction relationships were downloaded from Pfam to test this logic into the context of the GSP and GSN sets [76].

##### Cross-species interolog mapping

The human orthologs of model organism proteins often retain similar function; therefore, pair of human orthologs that interact in a model organism are likely to interact in human. Model organisms [Caenorhabditiselegans (4,649), Drosophila melanogaster (5,527), Saccharomyces cerevisiae (2,154), Rattusnorvegicus (15,306) and Musmusculus (16,376), Escherichia coli (541)] were mapped into human protein pairs, by using gene orthologs defined in the Inparanoid database by clustering into orthologous groups.

### Smallest shared biological process (SSBP)

Interacting proteins are usually involved in the same biological process; therefore, it is more likely to interact each other between the proteins functioning in small, specific processes. Functional similarity between two proteins was calculated according to following methods: 1) to screen all biological process involving two proteins shared; 2) to find how many other proteins in every shared process; 3) to determine the shared biological process with the fewest associated proteins. In general, the fewer proteins involved in the shared biological process indicate the greater functional similarity between two proteins. Protein pairs were determined by SSBP and then the degree of similarity was used to predict PPIs.

### Integration of different biological data into Naïve Bayesian model

A Naive Bayesian model was developed to integrate diverse data and make the final interaction predictions in an integrated way[77]. Following the Bayesian theorem, the posterior odds given n evidence as were computed as follows:

$$O_{posterior}=\frac{P(positive|E_{1},\ldots ,E_{n})}{P(negative|E_{1},\ldots ,E_{n})}$$

Where positive means that two proteins are functional related while negative means not. We define

$$LR_{\left(E_{1},\ldots ,E_{n}\right)}=\frac{P(E_{1},\ldots ,E_{n}|positive)}{P(E_{1},\ldots ,E_{n}|negative)}$$

then Oposterior = Oprior*LR. As

$$LR_{\left(E_{1},\ldots ,E_{n}\right)}=\prod_{i=1}^{n}LR_{\left(E_{i}\right)}$$

Naive Bayesian model supposes that each of the evidence is conditional independent, we can simplify LR as Since the prior odds is a constant, the predictive power or confidence degree for predicting functional links can be calculated by the composite LR corresponding to a type of specific biological evidence. A cutoff of likelihood ratio (LR cut) is represented as an indicator whether a protein pair bears the functional relation. Then, we filter the initial networks through Naïve Bayesian model by selecting the pairs with composite LR above the cutoff.

### Evaluation of Naive Bayesian network model

A receiver operating characteristic (ROC) curve can elucidate the relationship between the sensitivity and specificity of a binary classifier system for different cut points[78]. The ROC curve can be indicated equivalently by plotting the fraction of true positive rate (TPR) *versus* the fraction of false-positive rate (FPR). In a test, the ability of a classifier to identify true positives and false positives can be estimated by sensitivity and specificity, and calculated as sensitivity = TP/positives, and specificity = 1 - (FP/negatives), where TP and FP are the number of true positives and false positives identified by a classifier, respectively; whereas positives and negatives are the total number of positives and negatives in a test. The area under the ROC curve is used to assess the efficacy of the assessment system. Thus, the performances of different classifiers appear to be comparable by measuring the ROC curves, suggesting that the larger the ROC curve is; the better the performance is.

### Data analysis and statistics

Unpaired t-test or Pearson’s correlation test was used to compare quantitative variables; Patients’ survival curve was plotted by the Kaplan-Meier method, and the log-rank test was used to determine the significant difference among groups; the Cox regression model was used to perform multivariate analysis. Linear regression was tested by using the Spearman rank correlation. P <0.05 was considered statistically significant.

## SUPPLEMENTARY MATERIAL AND FIGURES


