# Supplementary material for: FGF8 promotes colorectal cancer growth and metastasis by activating YAP1
Source: Oncotarget. 2014 Nov 26;6(2):935–52. doi: 10.18632/oncotarget.2822 (PMC4359266; doi:10.18632/oncotarget.2822)
Supplement: Supplementary file 1 [file oncotarget-06-935-s001.pdf]

FGF8 promotes colorectal cancer growth and metastasis by activating YAP1

Supplementary Material

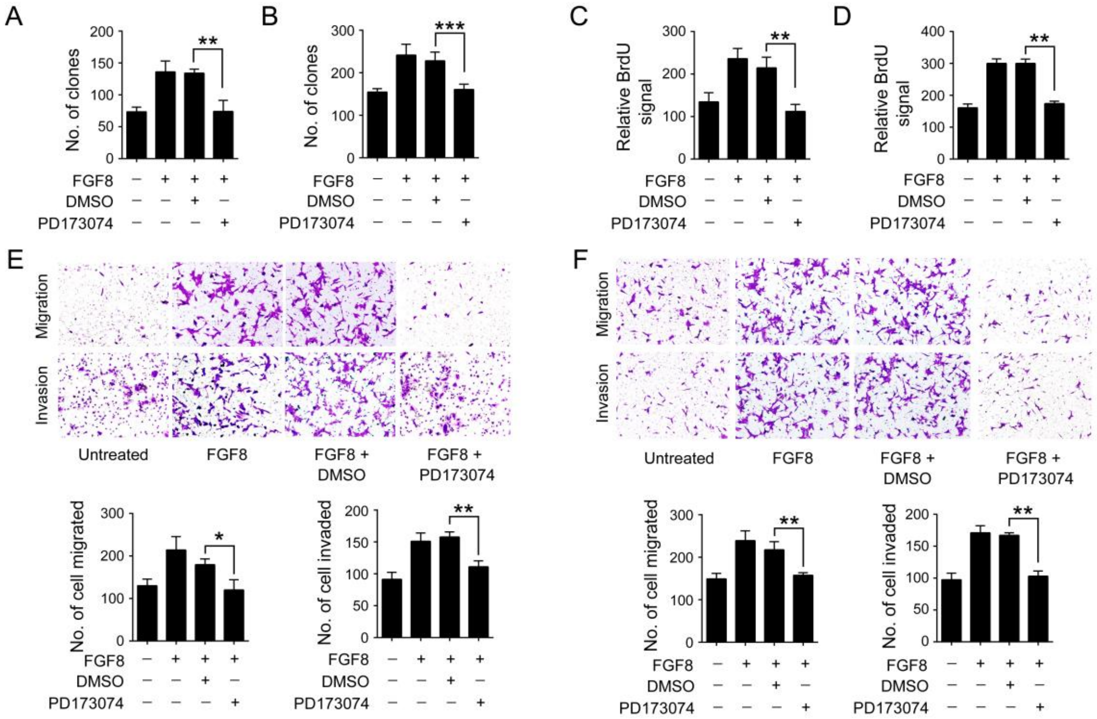

Figure S1: FGF8 promotes an aggressive phenotype in SW480 and HCT116 cells

SW480 and HCT116 cells were treated with or without FGF8 and/or PD173074. **(A-B)** Representative colony formation 14 days after culture of SW480 (A) and HCT116 (B) cells. **(C-D)** Proliferation rate of SW480 (C) and HCT116 (D) cells as measured by BrdU labeling for 12 hours. **(E-F)** Migration and invasion of SW480 (E) and HCT116 (F) was examined by transwell assay and matrigel invasion assays. Migration was analyzed at 24 h, invasion at 48 h. All data were from at least three independent experiments. *, P＜0.05; **, P＜0.01.

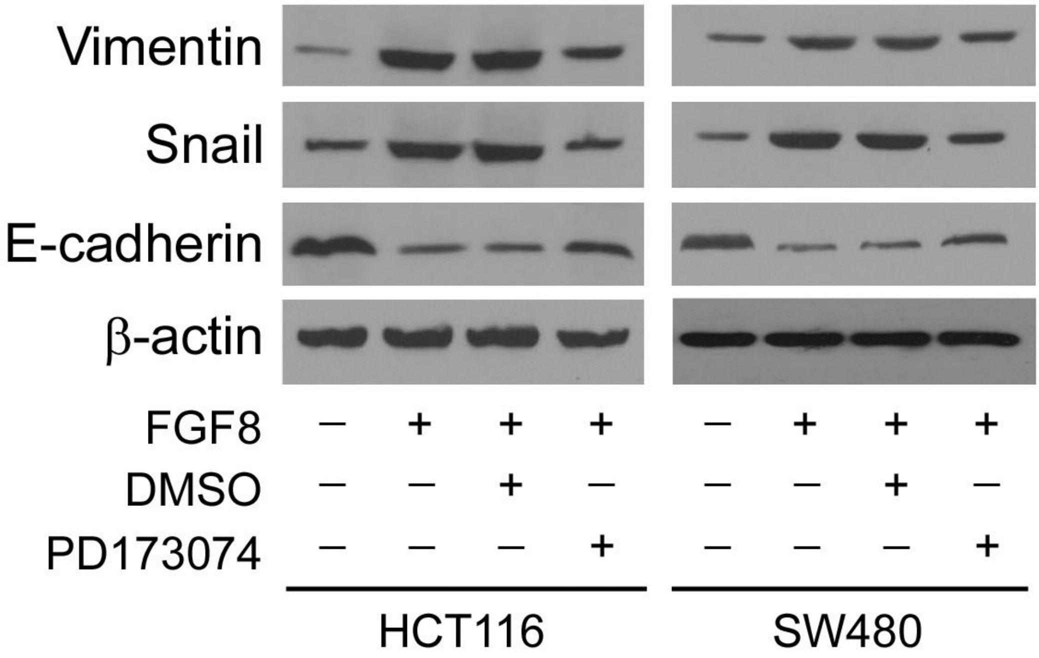

Figure S2: FGF8 induces EMT in SW480 and HCT116 cells

Expression of Snail, E-cadherin and Vimentin was examined by immunoblotting in SW480 and HCT116 cells treated with or without FGF8 and/or PD173074.

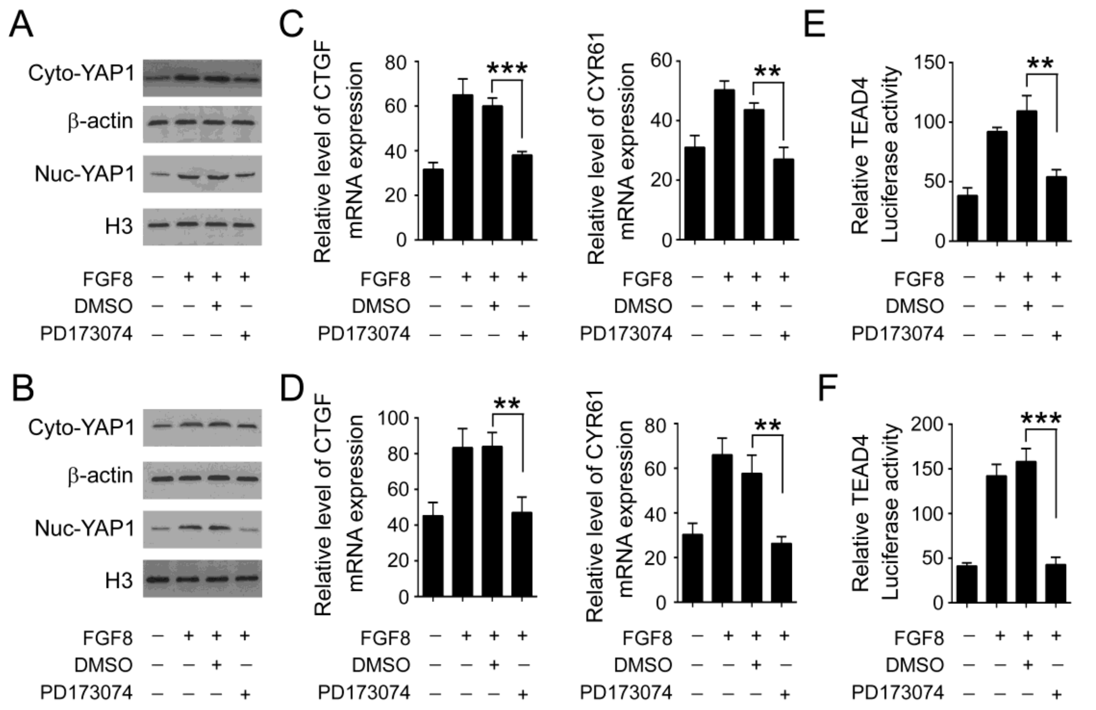

Figure S3: FGF8 can activate YAP1 signaling in SW480 and SW620 cells.

(A-B) Immunoblot analysis of expression of cytoplasmic and nuclear YAP1 in SW480 (A) and HCT116 (B) cells treated with or without FGF8 or/and PD173074. β-Actin was used as a cytoplasmic protein loading control, and histone-3 (H3) was used for nuclear protein loading control. **(C-D)** mRNA level of CTGF and CYR61 in SW480 (C) and HCT116 (D) cells treated with or without FGF8 or/and PD173074 was measured by qRT-PCR. **(E-F)** Transcription activity of TEAD4 in SW480 (E) and HCT116 (F) cells treated with or without FGF8 or/and PD173074 was measured by luciferase assay. All data were from at least three independent experiments. *, $P < 0.05$; **, $P < 0.01$; ***, $P < 0.001$.

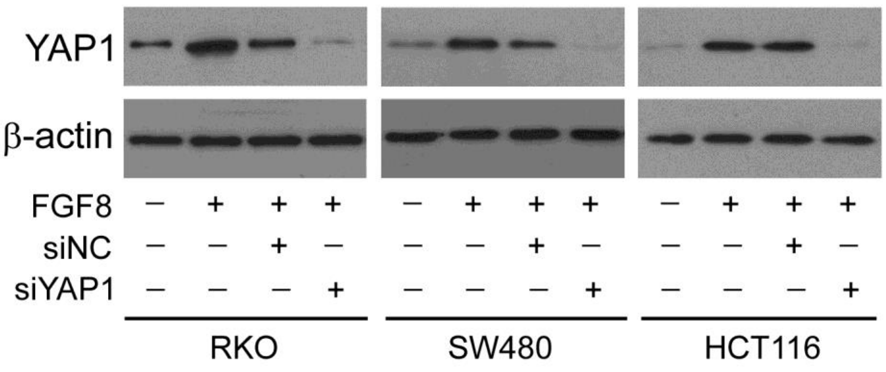

Figure S4: FGF8–induced expression of YAP1 was inhibited by siYAP1

Expression of YAP1 was examined by immunoblotting in FGF8-treated RKO (left) SW480 (middle) and HCT116 (right) cells transfected with siYAP1 or siNC.

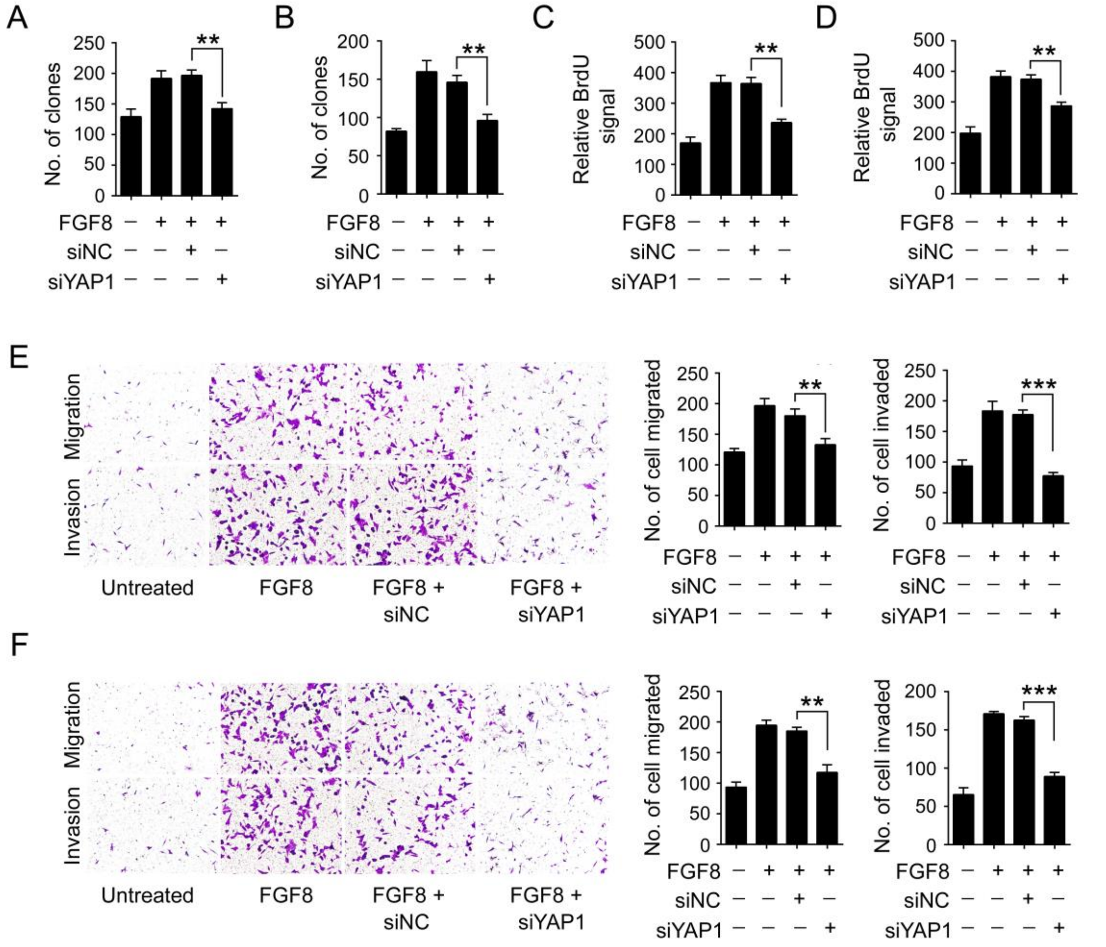

Figure S5: YAP1 is essential for FGF8-mediated SW480 and HCT116 tumor cell growth and metastasis

FGF8-treated SW480 and HCT116 cells were transfected with siYAP1 or siNC. **(A-B)** Proliferative activity of SW480 (A) and HCT116 (B) cell was measured by a colony formation assay. **(C-D)** Proliferative activity of SW480 (C) and HCT116 (D) cell was measured by BrdU labeling assay. **(E-F)** Migration and invasion of SW480 (E) and HCT116 (F) cell was examined by transwell assay and matrigel invasion assays. Migration was analyzed at 24 h, invasion at 48 h. All data were from at least three independent experiments. **, $P < 0.01$; ***, $P < 0.001$.

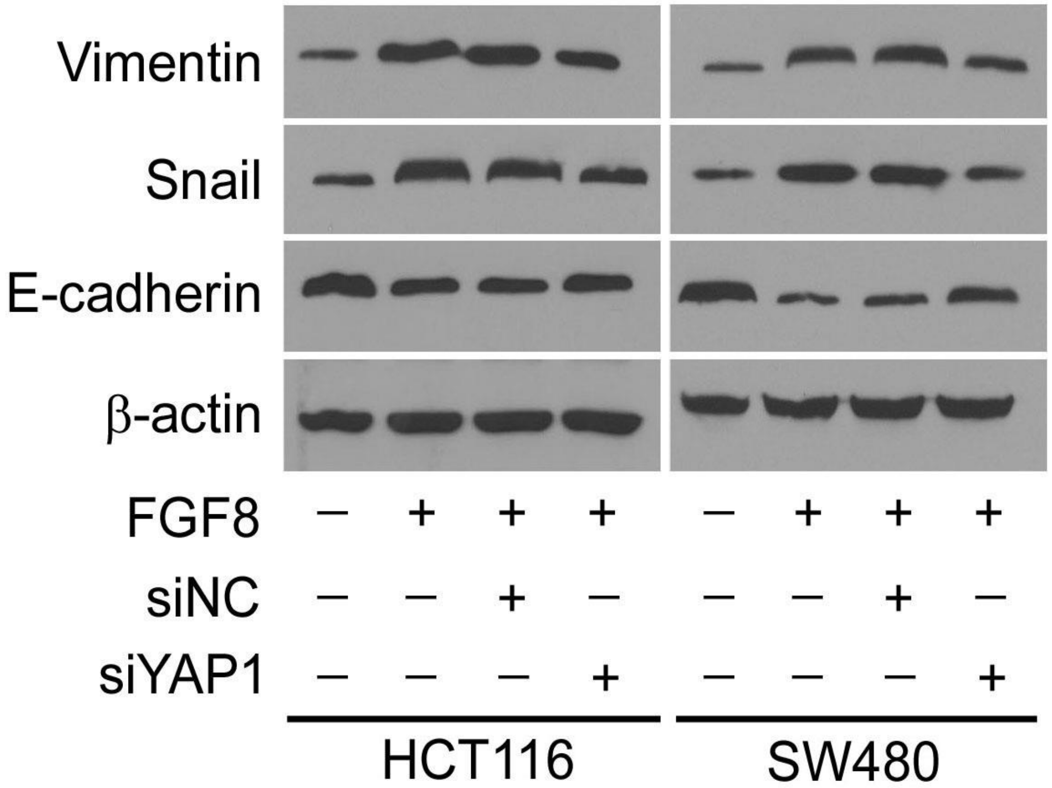

Figure S6: YAP1 is essential for FGF8-induced EMT in SW480 and HCT116 cells

Expression of Snail, E-cadherin and Vimentin was examined by immunoblot in FGF8-treated HCT116 (left) and SW480 (right) cells transfected with siYAP1 or siNC.

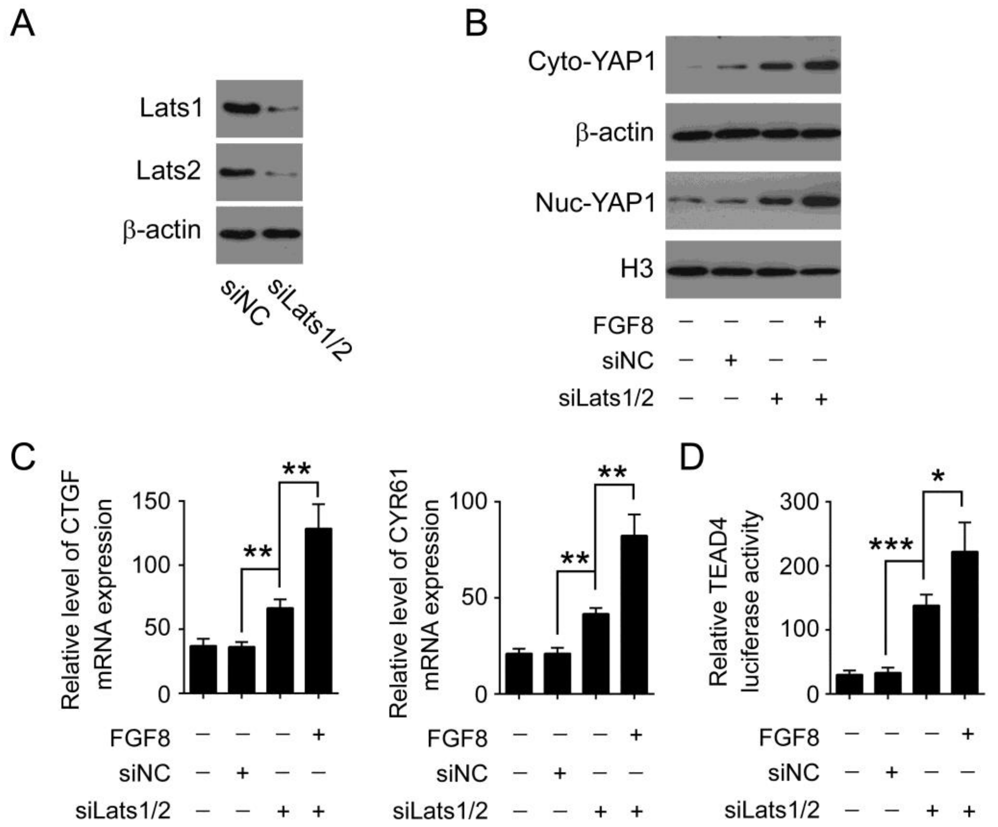

Figure S7: Hippo-independent pathways are involved in FGF8-induced YAP1 activation

(A) Expression of Lats1 and Lats2 was inhibited by siLats1/2 in RKO cells. **(B)** Immunoblot analysis of cytoplasmic and nuclear YAP1 in RKO cells treated with FGF8 or/and siLats1/2.β-Actin was used as a cytoplasmic protein loading control, and histone-3 (H3) was used for nuclear protein loading control. **(C)** The mRNA level of CTGF and CYR61 in RKO cells treated with FGF8 or/and siLats1/2 was analyzed by qRT-PCR. **(D)** Transcription activity of TEAD4 in RKO cells treated with FGF8 or/and siLats1/2 was analyzed by luciferase assay. All data were from at least three independent experiments. *, $P<0.05$; **, $P<0.01$; ***, $P<0.001$.
